# Guidelines on superficial soft tissue tumors: should they be revised?

**DOI:** 10.1186/s12885-025-15247-w

**Published:** 2025-12-01

**Authors:** Jun Guang Kendric Tan, Huayi Huang, Daniel Lee Jia Wei, Ruwan Wijesuriya

**Affiliations:** St John of God Midland Hospital, 1 Clayton St, Midland, WA 6056 Australia

**Keywords:** Lipoma, Sarcoma, Soft tissue lesion, Ultrasonography, Magnetic resonance imaging

## Abstract

**Background:**

Sarcomas guidelines suggest soft tissue lumps ≥ 5 cm, enlarging, painful or deep are considered malignant unless proven otherwise, should undergo a magnetic resonance imaging (MRI) scan and be referred to a specialist centre. Secondary hospitals receive multiple referrers from primary care for workup of subcutaneous, soft tissue lesions ≥ 5 cm with no other high-risk features. Strict adherence to recommendations can lead to overutilisation of limited resources.

**Methods:**

We performed a single centre, retrospective cohort study at St John of God Midland Hospital in Western Australia, Perth on 552 patients investigated for subcutaneous, soft tissue lesions from 24 November 2015 to 30 September 2024.

**Results:**

83.5% (461/552) of lesions assessed to be overall low-risk were excised locally. 31.9% (147/461) had ≥ 1 high-risk clinical feature but none were atypical or malignant. Histological lipomas were the most common at 83.5% (385/461) followed by angiolipomas at 9.1% (42/461). 0.22% (1/461) lesion showed malignancy and was < 5 cm. Pre-operative ultrasound was most commonly utilised at 56.2% (259/461) with 95.3% sensitivity and 100% specificity for lipomas. MRI was the next most commonly used imaging at 19.1% (88/461). 16.5% (91/552) of lesions had radiological high-risk features. 54.9% (50/91) were managed at our state sarcoma unit. 84% (42/50) underwent excisions with 1 histological pleomorphic sarcoma which had typical clinical and imaging features of malignancy managed with wide excision.

**Conclusion:**

99.8% (460/461) of patients with low-risk lesions <5cm were benign on histopathology. 50% (1/2) of subcutaneous, soft tissue lesions with malignancy were <5cm. Ultrasonography is highly sensitive (95.3%) and specific (100%) in characterising lipomas. This demonstrates that size alone should not be the sole indicator for escalation in investigation modalities (e.g. MRI), and that clinical assessment combined with ultrasonography is adequate in identifying low-risk lesions suitable for excision at non-sarcoma centres.

## Introduction

Sarcomas are a rare and diverse group of cancers arising from connective tissue, with an age-standardised incidence rate of 8 cases per 100,000 persons in the Australian population for 2018 [1]. Due to their infrequent presentation, they are historically poorly appreciated and can lead to delayed diagnosis with poor outcomes [2].

Current sarcoma literature focuses on epidemiological and contemporary description of sarcomas (annual incidence, prevalence, survival rate, treatment outcomes) [3–5]. Most published datasets originate from high volume tertiary sarcoma units where there is likely selection bias towards high-risk lesions [6], giving rise to soft tissue malignancy rates being reported at around 20% [6, 7]. Key recommendations suggests that any soft tissue lumps greater than 5 cm, are increasing in size, are deep to the deep fascia or painful to be considered malignant unless proven otherwise and referred to a specialist centre, with magnetic resonance imaging (MRI) being the preferred imaging modality [8, 9], likely stem from such statistics. In recent years, there has been recognition in the effectiveness of ultrasonography in differentiating benign versus malignant lesions. This is first reflected in the 2015 UK guidelines [10, 11] where ultrasonography is recommended as the initial triaging tool for any unexplained lump increasing in size. However, 2021 European guidelines still recommend MRI as the main imaging modality [8].

Despite this, there is a paucity of research data from peripheral hospitals. Secondary hospitals receive many referrers from primary care for workup of subcutaneous, soft tissue lesions greater than 5 cm with no other high risk features other than size. We also know from current literature that majority of soft tissue lesions will be subcutaneous and not deep-seated lesions [12]. Performing MRI scans and referring this entire patient cohort to specialised sarcoma centres for assessment can lead to overutilisation of a limited resource. Given that the bulk of elective excisions are performed by peripheral hospitals where their main role lies in triaging the large number of soft tissue lesions referred in by primary care, we believe there is a need for accurate representation of the landscape of superficial soft tissue lesions managed by peripheral hospitals.

We performed a retrospective review of all superficial soft tissue lesions managed at our centre to define the clinical characteristics of soft tissue lesions excised, provide an overview of the histological characteristics post-excision, correlating them with high-risk clinical features, reviewing the type of imaging utilised in pre-operative assessment and their concordance with histology.

We aim to accurately characterise the type of superficial soft tissue lesions in our local demographic and illustrate our centre’s local practices and outcomes. Using this data, we hope to show that subcutaneous, soft tissue lesions greater than 5 cm with no other high risk clinical features (other than size), with pre-operative ultrasonography not detecting any overt high risk radiological features, can safely proceed to surgical excision without MRI scans or further referral to a sarcoma unit.

## Methods

### Study Design

This retrospective cohort study was undertaken at St John of God (SJOG) Midland Public and Private Hospital, a 307-bed secondary public hospital in Western Australia, Perth. We captured all patients investigated for subcutaneous, soft tissue lesions by the general surgical department at SJOG Midland Hospital from 24 November 2015 to 30 September 2024.

SJOG Midland’s Theatre Management System (TMS) was interrogated for procedures linked to Medicare Benefits Schedule (MBS) item numbers 31,227, 31,345, 31,344, 31,220, 31,225, 30,611. Clinical data management systems CIS and Infomedix were interrogated for patient demographics; clinical history and examination details; biochemistry; operative note; imaging report and histopathological results. This yielded us 502 patients who had low risk lesions deemed appropriate for excision at our hospital.

However, this figure only captures patients who were operated at our hospital. To obtain the true number of soft tissue lesions in our catchment area, we obtained the number of referred patients that were not operated at our hospital. A request to Perth Radiological Clinic (PRC), a private on-site radiological provider attached to SJOG Midland, was also placed to access all ultrasound [8], computed tomography (CT) and magnetic resonance imaging (MRI) scans performed to investigate soft tissue lesions. This ensured patients who were investigated at our hospital but deemed high risk, unsuitable for excision at SJOG Midland and therefore referred to tertiary hospitals were captured in our study, giving a true representation of soft tissue lesions in our hospital’s catchment area. This analysis revealed 91 patients had high-risk clinical and radiological features There were all referred to our local sarcoma centre for further management. As part of this additional analysis, we also identified 315 patients with low-risk lesions who had their imaging performed at PRC but underwent further clinical care at hospitals external to us. This preventing interrogation of clinical records and hence were excluded for purposes our study.

Clinical high-risk features are defined as a rapid increase in lesion size, size larger than 5 cm, lesion that is non-mobile with underlying tethering and associated pain. Radiological high-risk features are defined as having focal or abundant vascularity, deep location, rapid interval growth, focal hyperechoic areas or heterogeneity, septations and consultant radiologist’s opinion on the overall lesion appearance [13].

Exclusion criteria included individuals less than 18 years old, soft tissue lesions deep to the subcutaneous layer and missing clinical details.

### Data collection

Clinical notes from SJOG Midland Hospital were reviewed by three investigators. Information collected included patient demographics; details of clinical presentation; operative note; imaging and histopathological results.

Quota sampling with specific characteristics defined by inclusion/exclusion criteria was used. Soft tissue sarcoma is an uncommon disease process and convenience sampling allows us the largest study population for increased study precision.

Self-selection bias is mitigated as our study population includes all patients investigated for soft tissue lesions from 24 November 2015 to 30 September 2024 at our centre. As our project is a retrospective cohort study based on chart review, non-response bias and under-coverage bias are minimised.

There will be minimal recall bias as all medical reports and letters interrogated are generated at time of procedure with objective endpoints. Sarcomas are usually a slow progressing pathology. We do not expect attrition from death of patients within the timeframe of our project.

During the course of data analysis, we noted about 90% of high-risk lesions picked up by radiology were referred directly onto tertiary hospitals, bypassing the surgical team at Midland. Due to differing clinical data management systems, we were unable to obtain details on their lesion’s clinical characteristics. Combining datasets will lead to data attrition bias. Hence, we decided to separate the analysis of low and high-risk lesions.

### Statical analysis

Comparative statistics were performed with a two-tailed Chi-squared test. A p-value of < 0.05 was considered statistically significant.

### Ethics

Local ethics approval was obtained through the St. John of God Health Care Human Research Ethics Committee (approval number: 2237). All data was de-identified, password protected and kept on secure servers.

## Results

A total of 593 patients were investigated for subcutaneous, soft tissue lesions at our centre. 41 were excluded due to a lack of histology results and clinical details, bringing our study population down to 552. The median age of all patients was 55 (range 18–90), with a female to male ratio of 1.35 : 1 (Male: 317, Female: 235).

### Clinical characteristics of lesions

**Fig. 1:**
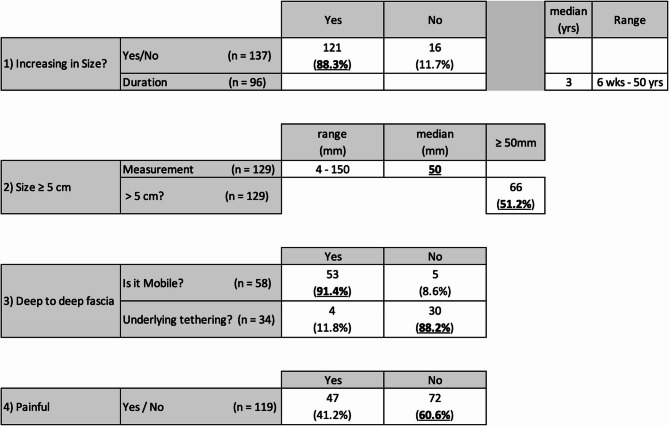
Clinical characteristics of soft tissue lesions excised at SJOG Midland from 2017–2024

83.5% (461/552) of patients had lesions excised at our center. Of the 83.5% (461/552), 44.0% (203/461) had clinical notes available to extract clinical characteristics of lesions documented during outpatient clinic evaluation. 88.3% (121/137) of patients reported their lesion to have increased in size, with median duration over 3 years (6 weeks – 50 years). 51.2% (55/129) of lesions were measured to be greater than 50 mm, with a median size of 50 mm (4–150 mm). 8.6% (5/58) of lesions were mobile and 11.8% (4/34) had underlying tethering. 41.2% (47/119) were reported as painful. Of the 147 lesions with one or more clinically high-risk features, none were atypical or malignant on histopathological examination. This is illustrated in Fig. 1.

### Imaging modality

**Fig. 2:**
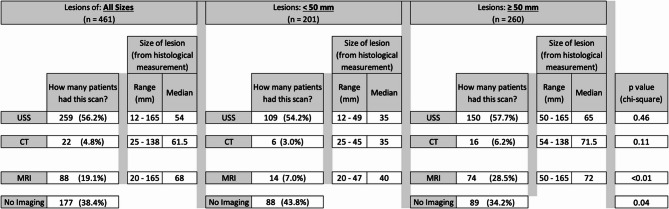
Imaging modality utilised in the workup of soft tissue lesions excised at SJOG Midland from 2017–2024

For lesions of all sizes, 56.2% (259/461) were investigated pre-operatively with ultrasonography [8], 4.8% (22/461) with computer tomography (CT) scans and 19.1% (88/461) with magnetic resonance imaging (MRI). When looking at lesions smaller than 50 mm, 54.2% (109/201) underwent USS, 3% (6/201) underwent CT and 7% (14/201) underwent MRI scans. 38.4% (177/461) lesions underwent no imaging. For lesions larger than 50 mm, 57.7% (150/260) underwent USS, 6.2% (16/260) underwent CT and 28.5 (75/260) underwent MRI scans. 34.2% (89/260) lesions underwent no imaging. This is illustrated in Fig. 2.

### Overview of histology

**Fig. 3:**
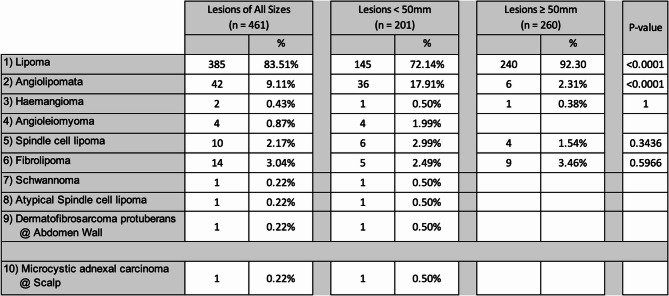
Overview of histology of soft tissue lesions excised at SJOG Midland from 2017–2024

Figure 3 shows the breakdown of histology for lesions excised. The most common histological finding for lesions of all sizes was lipomas at 83.5% (385/461). This was divided into 72.1% (145/201) for lesions less than 5 cm, and 92.3% (240/260) for lesions greater than 5 cm. The second most common lesion was angiolipomas at 9.1% (42/461), distributed into 17.9% (36/201) for lesions less than 5 cm, and 2.3% (6/260) for lesions greater than 5 cm. 0.22% (1/461) lesion was malignant, measuring less than 5 cm.

### Corelation of operative histology with pre-operative imaging

Comparing operative histology with pre-operative imaging findings, 99.5% (210/211) of USS and 97.4% (75/77) of MRI findings were in concordance with final histological results. Angiolipomas were not reliability differentiated from lipomas with USS, and only 25% (1/4) was identified on MRI. Angioleiomyoma, spindle cell lipomas and fibrolipoma were all not differentiated on pre-operative imaging.

### Sensitivity and specificity of imaging

USS was 95.3% sensitive for lipomas with 100% specificity. Positive predictive value (PPV) was 100%, with negative predictive value (NPV) of 7.69%. MRI was 94.1% sensitive for lipomas with 100% specificity. Positive predictive value (PPV) was 100%, with negative predictive value (NPV) of 37.5%.

### Management of lesions with radiologically high-risk characteristics

16.5% (91/552) of all patients investigated at our centre had high risk features seen on imaging. Of the 10 ultrasound scans, 10% (1/10) had cystic changes, 20% (2/10) were heterogenous in appearance, 30% (3/10) was classified high risk based on size ≥ 5 cm alone and 40% (4/10) were vascular. Of the 78 MRI scans, 46.2% (36/78) was classified high risk based on size ≥ 5 cm alone, 29.5% (23/78) were heterogenous in appearance, 11.5% (9/78) had significant enlargement on interval imaging, 9.0% (7/78) were septated, 2.6% (2/78) invaded into surrounding structures, and 1.3% (1/78) had fat necrosis. Of the 2 CT scans, 50% (1/2) showed complexity and 50% (1/2) was classified high risk based on size ≥ 5 cm alone. 1 FDG PET showed the lesion to be PET avid.

54.9% (50/91) were referred to Western Australia’s public state sarcoma unit for further management. 45.1% (41/91) had no publicly accessible records and assumed to have been referred to the private sector for management. Of the 50 patients managed publicly, 84% (42/50) underwent surgical excision. There was one case of undifferentiated pleomorphic sarcoma (2.4%, 1/42) which underwent wide excision, with the rest being lipomas (52.4%, 22/42), spindle cell lipoma (9.5%, 4/42), hibernoma (2.4%, 1/42). 33.3% (14/42) had no histology available for our analysis. The remaining 16% (8/50) who did not undergo surgical excisions either had core biopsies showing benign lesions, were placed on surveillance plans or pending multidisciplinary meeting consensus.

## Discussion

To our knowledge, this is the first study on soft tissue lesions managed in a Western Australian peripheral hospital. Our study revealed for all subcutaneous, soft tissue lesion of all sizes investigated in the setting of a secondary, 307-bed public hospital in Western Australia, Perth, the overall malignancy rate for all lesions was 0.36% (2/552). Ultrasonography was highly accurate at characterising lesions with a 99.5% (210/211) concordance rate with final histopathology. 66.7% (2/3) of lesions which were malignant were smaller than 5 cm and would not have been identified based on size criteria for high-risk lesions. It is important to note that our study focuses only on subcutaneous lesions and not deeper lesions. This accurately represents lesions being excised in peripheral hospitals, and thus underscores the novelty of our study, but may underestimate overall rates of soft-tissue malignancy.

The assessment of soft tissue lesions begins in the outpatient setting where clinicians risk-stratify lesions based on clinical characteristics. Most patients seek medical attention when their lesion either becomes painful or grows to a size where it is bothersome in their activities of daily living. This is reflected where 88.3% (121/137) of patients reported their lesion to have increased in size and 41.2% (47/119) reported pain. Painful lesions tended to be angiolipomas at 53.3% (8/15), whereases simple lipomas are only painful in 36.5% (35/96) of cases. Published guidelines do not suggest a time frame defining ‘increasing in size’. However, rapid size increase in weeks should raise concerns over one present for years. In our study, the only malignant lesion greater than 5 cm had rapid increase in size over the course of 6 weeks, flagging it for potential malignancy. In the community, rather than defining high risk characteristics as ‘increasing in size’ or ‘greater than 5 cm’, definitions such as ‘rapid size increase over weeks to months’ might better appreciate such nuances. We also found the four clinical signs suggesting a high risk lesion [9] to have a low sensitivity in our patient cohort. Of the 203 patients with clinical notes, 72% (147/203) patients had one of more red flags. However, none of them had histological atypia or malignancy. Of these 147 patients, 74% (110/147) were investigated with USS, 39% (58/147) with MRI and 6% (9/147) with CT scans. Hence, we believe despite the presence of traditionally defined high risk physical examination findings, if lesions are corelated with appropriate imaging and access to multidisciplinary sarcoma services, excisions can be safely performed at peripheral centres.

Over the course of 9 years, 461 lesions were excised at SJOG Midland. 56% (260/461) of all lesions were ≥ 5 cm per histological measurement, reinforcing our belief that the majority of large soft tissue lesions are managed in a peripheral setting rather than tertiary specialist centres. None of these lesions were atypical or malignant on histology. For lesions of all sizes, USS was the most commonly used imaging modality at 56% (259/461). We know USS has high sensitivity and specificity in the evaluation of superficial soft tissue masses [14, 15], and society consensus statements reinforce the suitability of USS as first line imaging in the evaluation of such lesions [13]. The second most used imaging modality was MRI scans at 19.1% (88/461). MRI scans are usually suggested by radiology when a lesion fails to demonstrate characteristic or pathognomonic features on USS that enable a confident diagnosis. Potential advantages of MRI scan include allowing better appreciation of surrounding structures (musculoskeletal, neurovascular), assessing deep lesions where transducer access is limited and surgical planning [16]. T1-weighted and a fluid-sensitive, fat-saturated sequence is mandatory for MRI scans investigating soft tissue lesions [17]. However, Goldman et al. demonstrated that for lesions that were non-diagnostic on USS, MRI scans does not increase the specificity regarding tumor type. MRI scan was not diagnostically useful in 68% of cases, and did not change the working diagnosis in 73% of cases [18]. Analysing the indications for MRI scans, we found the majority to be requested by radiology in ultrasound reports based on size criteria alone. This is reflected where MRI scans are used more commonly in lesions ≥ 5 cm at 28% (75/260) compared to 7% (14/201) for lesions < 5 cm. Lesions that were histologically < 5 cm had MRI scans generally because other imaging modalities such as USS or CT scan showed the lesion either to be >5 cm radiologically or having atypical features. CT scans are least commonly used at 19.1% (88/461) due to its limited ability to assess soft tissue. It is only useful when lesions are either deep seated, or around vascular structures (e.g. neck) to assist operative approach.

Analysing the histology of lesions excised, over 92% (427/461) of all lesions were either simple lipomas or angiolipomas. Majority of lipomas present when ≥ 5 cm (92.3%, 240/260), rather than < 5 cm (72.1%, 145/201, *P* < 0.01). This is representative of the painless nature of simple lipomas, where the impetus for patients presenting usually revolves around progressive interference with daily activities. However, patients with angiolipomas mainly present when < 5 cm (17.9%, 36/201), with few waiting for sizes ≥ 5 cm (2.3%, 6/260, *P* < 0.01). This is in keeping with the classic painful nature of angiolipomas [19] where patients seek medical attention at early stages of growth.

In terms of malignant lesions, of the 461 lesions excised at our hospital, there were one microcystic adnexal carcinoma. This lesion was < 5 cm, did not satisfy any high-risk clinical features and did not undergo pre-operative imaging. Historical studies by Johnson et al. quoted 70.5% of lesions ≥ 5 cm to be malignant, justifying that such lesions should be considered malignant until proven otherwise [20]. More recent data by Gassert et al. further explored superficial lesions ≤ 5 cm and found 1 in 4 to be malignant. In light of this, we believe malignant lesions are not always predictable despite risk stratification tools. Surgical excisions should be considered for all soft tissue lesions that are new, or has interval change in size.

The calculation of sensitivity and specificity depends on what can or cannot be differentiated on imaging, and if the differentiation is on a histological basis. For example, fibrolipoma and spindle cell lipoma are histological classifications which cannot be differentiated on imaging. Although there is some evidence that angiolipomas can be reliably identified on imaging [21], we believe pre-operative differentiation of such benign lesions to be of minimal clinical significance. Therefore, if we classify angiolipoma, fibrolipoma and spindle cell lipoma as lipomas for the purposes of this calculation, we arrive at a sensitivity of 95.3% and specificity of 100% at USS identifying lipomas, in keeping with current literature [14, 15]. The combination of high sensitivities of USS and unpredictability of soft tissue cancers leads us to believe all lesions, regardless of clinical characteristics, should be assessed with a pre-operative USS prior to excision.

16.5% (91/552) of our patient cohort had imaging requested by referring physicians identifying high risk characteristics. These can include interval change in size compared with previous imaging, lack of similarity with the known USS appearances of particular benign soft tissue masses or disorganised vascular patterns [16]. This patient cohort was referred directly to Western Australia’s public state sarcoma unit for multidisciplinary discussion and management. 3 in 4 lesions were offered excisions. The remainder were either deemed low risk on imaging review and placed on surveillance or pending discussion. Of those excised, one patient had undifferentiated pleomorphic sarcoma of the anterior abdominal wall. This lesion was clearly clinically concerning, with rapid growth in weeks prior to presentation, measured 95 mm, had USS findings of a non-compressible, complex heterogenous solid mass with internal vascularity and MRI showing heterogeneous contrast enhancement central necrosis. These sonographic findings are in keeping with what is known in the literature, with typical findings for non-specific neoplastic masses to be solid or mixed solid/cystic structures, having variable heterogenicity and internal vascularity [15]. This was the singular lesion ≥ 5 cm with malignant histopathology in our study population.

Percutaneous core biopsies have a role in in establishing histopathological diagnosis for suspicious soft tissue masses [8, 10]. Recent studies demonstrated concordance of entity in up to 98% and subtype diagnosis in 87% of cases between pre-operative core needle biopsy and final operative histopathology [22]. Core biopsies are not currently offered in our secondary hospital as we only surgically manage low-risk lesions. Indeterminate or suspicious lesions are referred on to our state sarcoma unit for further management.

Looking at the sum of our data, we note the unpredictable clinical findings soft tissue cancers can present with, as well as the high sensitivity and specificity of USS in identifying benign soft tissue lesions. Hence, we believe all lesions should be investigated with a pre-operative USS to rule out high risk features. For lesions with normal USS findings but with size > 5 cm as the only clinical red flag, unless there is a clinical need to define exact tumor location or anatomy, MRI scans are not always necessary and rarely change management.

**Fig. 4:**
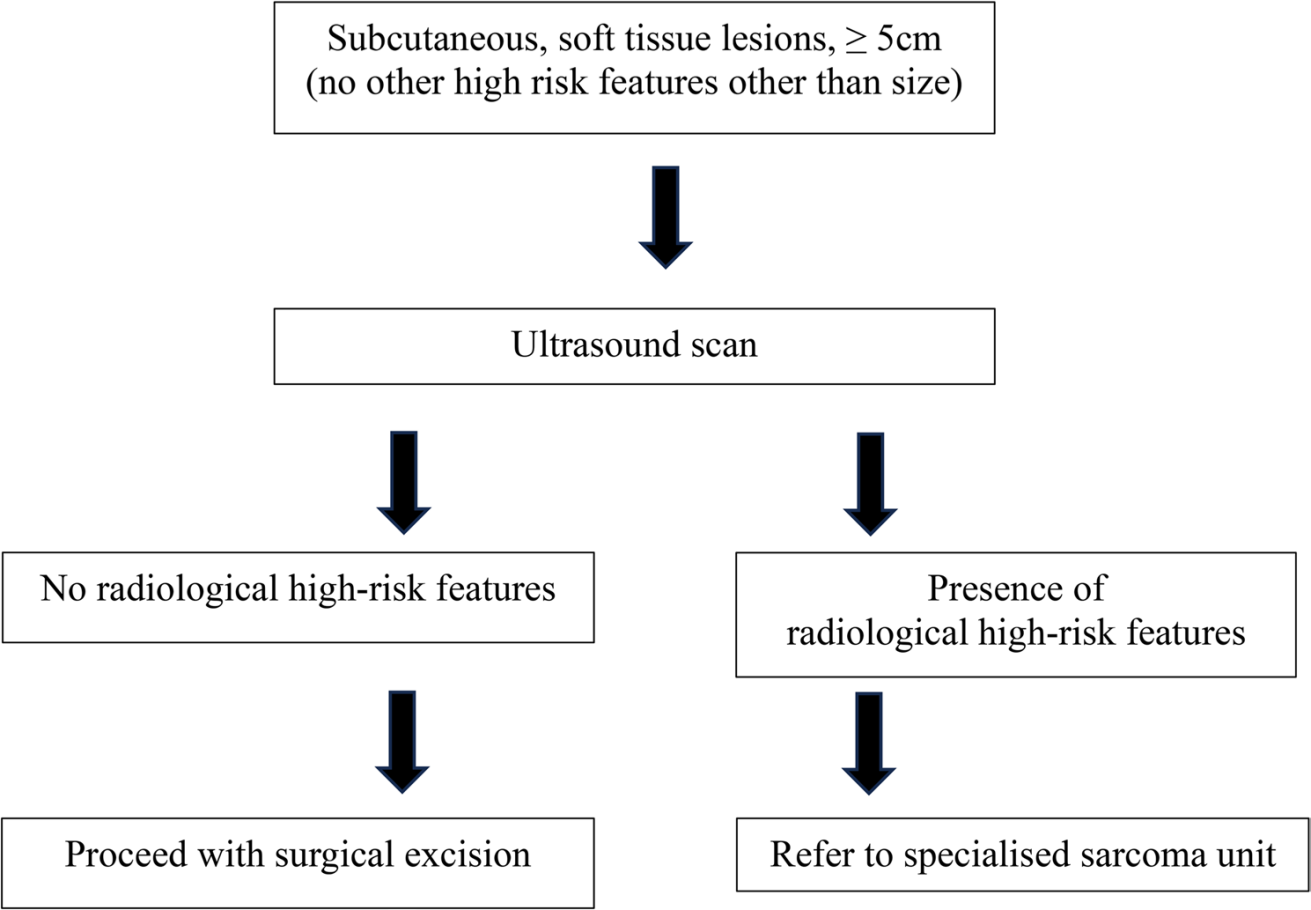
Proposed management algorithm of subcutaneous, soft tissue lesions, ≥ 5 cm (with no other high risk features other than size) in a centre without a specialised sarcoma unit

We also suggest surgical excisions be considered for soft tissue lesions that are new, or has interval change in size. It is not always possible to identify malignancy without excisional biopsies and as we demonstrated, 1 out of 2 lesions with malignancy were < 5 cm with no clinical red flags. Despite lesions having ‘high risk physical examination findings’, if corelated with appropriate imaging and performed in centres with access to multidisciplinary sarcoma services for second opinions, excisions can be safely performed at peripheral centres. In Fig. 4, we propose a simple management algorithm for subcutaneous, soft tissue lesions ≥ 5 cm (with no other high risk features other than size) in a centre without a specialised sarcoma unit.

It is prudent to note the high excision rates in our centre to be reflective of our local healthcare structure. Our patient cohort seek specialist attention for soft tissue lesions mainly for symptomatic relief. Being a secondary hospital, we have funding allowing for such procedures. For long standing subcutaneous lesions with no change in size, characteristics, no clinical red flags and ultrasonography showing bland lipomas, given the high sensitivity of USS, it is reasonable to reassure patients the benign nature of such lesions and manage them conservatively without surgical excision.

Limitations of our study include having relatively low rates of malignancy (0.40%, 2/511) in our study population. This limited our ability to robustly satisfy power calculations. In addition, a significant number of patients (56.0%, 258/461) did not have clinical notes available to extract clinical characteristics of lesions documented during outpatient clinic evaluation. This limited our ability to give a comprehensive overview of lesion characteristics. The retrospective nature of our study also meant we could not ensure all lesions had the recommended pre-operative imaging. However, we believe our study fills an important void in currently published literature. We give a true representation of how soft tissue lesions present in a peripheral centre rather than in a specialised, tertiary centre with selection bias towards high-risk lesions.

## Conclusion

Soft tissue lesions are very common in the community. Peripheral hospitals receive majority of such referrers and must strike a balance between patient safety and resource utilisation. Traditional teaching proposes a low threshold for malignancy and keenly suggests onward referral to specialised sarcoma units. We believe the landscape of soft tissue lesions seen in the community to be more benign, but somewhat more unpredictable than what early papers suggest. A measured approach combining thorough clinical assessment, mandatory assessment with ultrasonography and excising any growing lesion might offer a more balanced means of management. Recognition of red flags on pre-operative workup and intraoperatively (solid lesion, atypical tissue quality), together with robust histopathological services and timely review of reports are practices we should follow and confers a safety netting to atypical lesions.

We also make the case for reducing utilization of MRI scans and reducing onward referrals to sarcoma centres based on size criteria > 5 cm alone. With malignancy rates of low-risk soft tissue lesions < 5 cm at 0.22% (1/461) and sensitivity of USS at detecting lipomas at 95.3%, excisions can be safely performed at peripheral centres based on clinical assessment and USS alone.

Given the lack of similar studies in this underreported aspect of soft tissue lesions, further research in other comparable peripheral hospitals to evaluate homogeneity of our findings is warranted in both a national and international setting.

## Data Availability

The data that support the findings of this study are available from the corresponding author, Tan Jun Guang Kendric, upon reasonable request.
